# Organic–Inorganic Hybrid Perovskite Ferroelectric Nanosheets Synthesized by a Room‐Temperature Antisolvent Method

**DOI:** 10.1002/advs.202400636

**Published:** 2024-05-22

**Authors:** Tai‐Ting Sha, Xing‐Chen Zhang, Ru‐Jie Zhou, Guo‐Wei Du, Yu‐An Xiong, Qiang Pan, Jie Yao, Zi‐Jie Feng, Xing‐Sen Gao, Yu‐Meng You

**Affiliations:** ^1^ Jiangsu Key Laboratory for Science and Applications of Molecular Ferroelectrics Southeast University Nanjing 211189 P. R. China; ^2^ Guangdong Provincial Key Laboratory of Quantum Engineering and Quantum Materials and Institute for Advanced Materials South China Academy of Advanced Optoelectronics South China Normal University Guangzhou 510006 P. R. China

**Keywords:** antisolvent method, ferroelectric domains, ferroelectric nanosheets, molecular ferroelectrics, organic–inorganic hybrid perovskite

## Abstract

Over the past years, the application potential of ferroelectric nanomaterials with unique physical properties for modern electronics is highlighted to a large extent. However, it is relatively challenging to fabricate inorganic ferroelectric nanomaterials, which is a process depending on a vacuum atmosphere at high temperatures. As significant complements to inorganic ferroelectric nanomaterials, the nanomaterials of molecular ferroelectrics are rarely reported. Here a low‐cost room‐temperature antisolvent method is used to synthesize free‐standing 2D organic–inorganic hybrid perovskite (OIHP) ferroelectric nanosheets (NSs), that is, (CHA)_2_PbBr_4_ NSs (CHA = cyclohexylammonium), with an average lateral size of 357.59 nm and a thickness ranging from 10 to 70 nm. This method shows high repeatability and produces NSs with excellent crystallinity. Moreover, ferroelectric domains in single NSs can be clearly visualized and manipulated using piezoresponse force microscopy (PFM). The domain switching and PFM‐switching spectroscopy indicate the robust in‐plane ferroelectricity of the NSs. This work not only introduces a feasible, low‐cost, and scalable method for preparing molecular ferroelectric NSs but also promotes the research on molecular ferroelectric nanomaterials.

## Graphical abstract

This article presents a low‐cost room‐temperature antisolvent method to synthesize free‐standing 2D organic–inorganic hybrid perovskite ferroelectric nanosheets, that is, (CHA)_2_PbBr_4_ NSs (CHA = cyclohexylammonium). Ferroelectric domains in the NSs are clearly visualized and manipulated using piezoresponse force microscopy. This work not only introduces a feasible, low‐cost, and scalable method for preparing molecular ferroelectric NSs but also promotes the research on molecular ferroelectric nanomaterials.

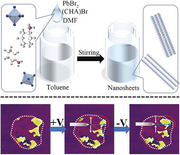

## 1 Introduction

Ferroelectrics are functional materials with spontaneous polarization that can be reversed by an applied electric field. These materials also exhibit piezoelectricity and pyroelectricity. Due to these features, ferroelectrics are widely used in capacitors, transducers, actuators, energy harvesting devices, and nonvolatile memories, particularly, in consumer electronics.^[^ ^1^, ^2^, ^3^, ^4^, ^5^, ^6^ ^]^ Traditional inorganic ferroelectrics and polymer ferroelectrics have always been a hot topic of research. Now similar concerns are being paid to molecular ferroelectrics, due to their simple fabrication, flexibility, diversity of compositions, biocompatibility, and semiconducting performance.^[^ ^7^, ^8^, ^9^, ^10^ ^]^ A typical example is the high‐performance OIHP‐type molecular ferroelectrics, such as TMCM‐CdCl_3_ (trimethyl chloromethylammonium cadmium trichloride)^[^ ^11^ ^]^ with a large piezoresponse (*d*_33_ = 383 pC/N), (cyclohexylammonium)_2_PbBr_4−4x_I_4x_ (x = 0−1)^[^ ^12^ ^]^ with an adjustable band gap in the range of 3.05−2.74 eV, and bis(4,4‐difluoropiperidinium) lead tetraiodide^[^ ^13^ ^]^ with eight‐fold vertex domains.

In modern electronics, ferroelectric nanomaterials are of great significance in device miniaturization. For example, the density of ferroelectric nonvolatile memories can be increased thousands‐fold by reading and writing in devices based on ferroelectric nanomaterials.^[^ ^14^, ^15^, ^16^ ^]^ Compared with their bulk materials, ferroelectric nanomaterials possess quite distinct properties due to their small size and large surface‐to‐volume ratios. Extensive studies on inorganic ferroelectric nanomaterials have promoted the discovery of many fascinating physical phenomena, such as the topological structure^[^ ^5^, ^17^, ^18^, ^19^, ^20^, ^21^, ^22^, ^23^, ^24^, ^25^ ^]^ and size effect.^[^ ^26^, ^27^, ^28^ ^]^ This has naturally made us ponder upon the great potential of their molecular counterparts, that is, molecular ferroelectric nanomaterials, which may exhibit distinctive features due to the unique polarization mechanism and structural variety.^[^ ^7^, ^8^, ^29^ ^]^ However, molecular ferroelectrics nanomaterials are rarely reported.

Of all ferroelectrics, traditional perovskite oxides^[^ ^30^ ^]^ are the most widely used types, whose nanomaterials are mainly fabricated by pulsed laser deposition (PLD),^[^ ^18^, ^24^, ^25^ ^]^ sol–gel technique,^[^ ^31^, ^32^, ^33^ ^]^ and hydrothermal synthesis.^[^ ^34^, ^35^ ^]^ The fabrication is primarily achieved at high temperature under vacuum.^[^ ^30^, ^36^ ^]^ During the past decade, 2D van der Waals (vdW) ferroelectric materials have emerged, which may open up opportunities for ferroelectric materials and devices.^[^ ^36^, ^37^, ^38^, ^39^, ^40^ ^]^ A simple and effective method to obtain the desired nano‐thickness films of vdW ferroelectrics is mechanical exfoliation,^[^ ^41^, ^42^ ^]^ which can be performed at room temperature and under ambient pressures. However, this method exhibits low scalability and usually produces NSs that are randomly sized and shaped.^[^ ^36^, ^43^ ^]^ Furthermore, ferroelectricity has been observed in fluorite‐structured oxides, thereby revitalizing the research interest in nanoscale ferroelectric memories.^[^ ^44^, ^45^, ^46^ ^]^ A typical method to fabricate fluorite‐structured oxide nanomaterials is the atomic layer deposition (ALD) technique,^[^ ^45^, ^47^ ^]^ which requires vacuum and the protection of inert gases.^[^ ^48^, ^49^ ^]^ The synthesis of molecular ferroelectrics through convenient solution methods^[^ ^9^, ^11^ ^]^ has motivated us to fabricate ferroelectric nanomaterials using a similar solution method. Inspired by the synthesis of lead halide perovskite photoluminescence nanosheets (NSs),^[^ ^50^, ^51^ ^]^ we adopt a feasible and low‐cost antisolvent method to synthesize free‐standing (CHA)_2_PbBr_4_ (CHA = cyclohexylammonium) NSs. The method, with high repeatability, can be performed at room temperature and under ambient pressure. Furthermore, the fabricated NSs show excellent crystallinity, with an average lateral size of 357.59 nm and a thickness ranging from 10 to 70 nm. In addition, the domain switching and PFM‐switching spectroscopy have confirmed the robust in‐plane ferroelectricity of the NSs. This work demonstrates a feasible, low‐cost, and scalable method for preparing OIHP ferroelectric nanomaterials, which promotes the research and applications of ferroelectric nanomaterials.

## 2 Results and Discussion

### 2.1 Preparation and Characterization of (CHA)_2_PbBr_4_ NSs

**Figure 1:**
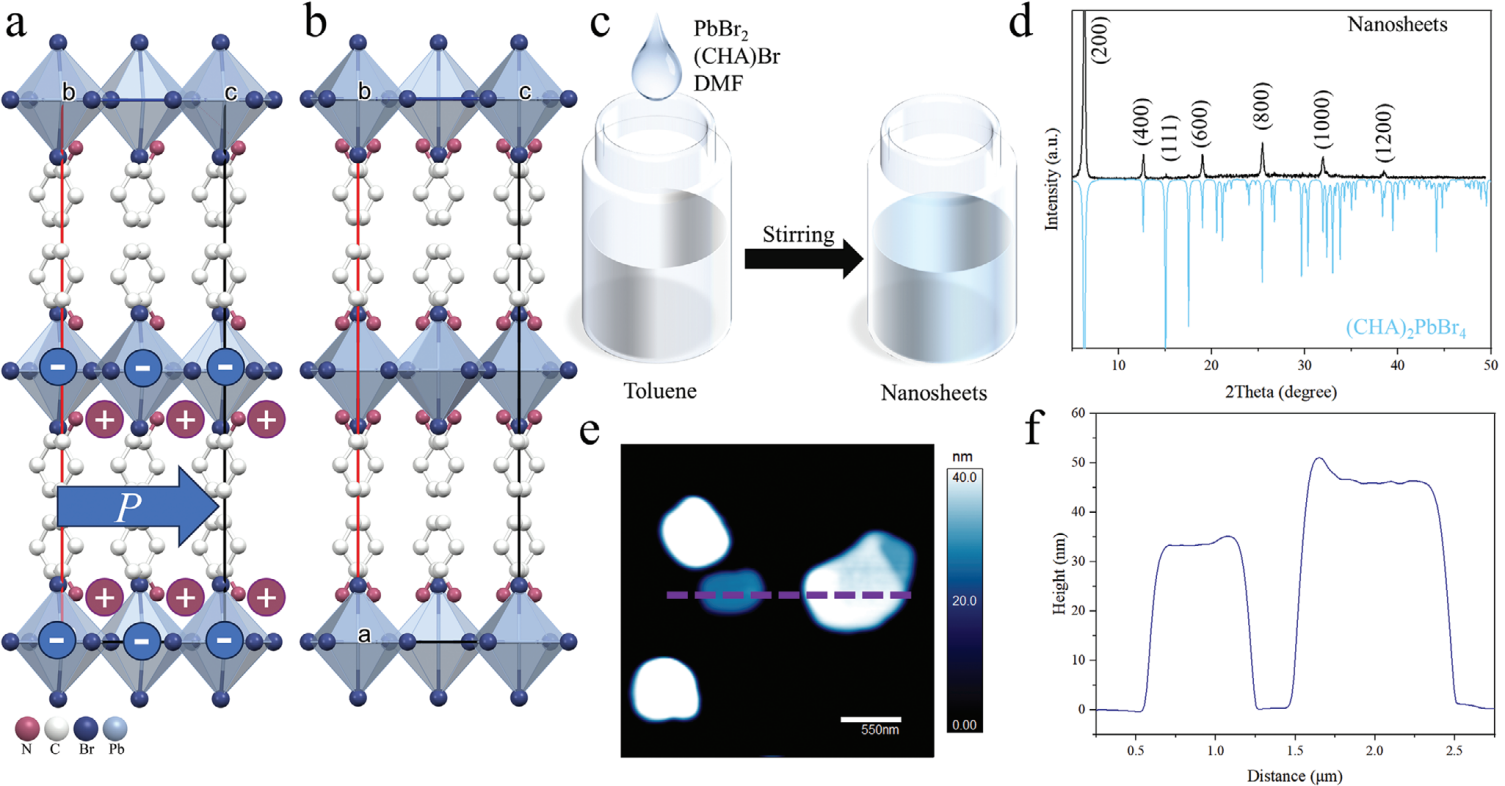
Crystal structure and characterizations of (CHA)_2_PbBr_4_ NSs. Crystal structures of the layered perovskite (CHA)_2_PbBr_4_ in a) ferroelectric and b) paraelectric phases. The blue arrow indicates the spontaneous electric polarization. The positive and negative charge centers are marked as + and −, respectively. c) Sketch of the synthesis of (CHA)_2_PbBr_4_ NSs. d) XRD patterns of the (CHA)_2_PbBr_4_ NSs and simulated patterns of (CHA)_2_PbBr_4_. e) AFM image of (CHA)_2_PbBr_4_ NSs. f) Height profiles along the dash lines denoted in (e).

As shown in **Figure** **1**a,b, (CHA)_2_PbBr_4_ is a typical 2D Ruddlesden–Popper (RP) type perovskite. The crystal structure of (CHA)_2_PbBr_4_ is characterized by infinite and staggered layers of PbBr_6_ octahedra sharing corners, with CHA cations interleaved between them.^[^ ^52^ ^]^ These large CHA cations not only enhance the colloidal stability of the nanostructures but also prevent the formation of a 3D framework. At room temperature (Figure 1a), this perovskite crystallizes in the polar space group *Cmc*2_1_, where all the C─N bonds of the CHA cations align along the c‐axis, inducing polarity.^[^ ^52^ ^]^ This arrangement results in a displacement of ≈0.82 Å of the positive charge carried by the nitrogen atoms, contributing to spontaneous polarization of ≈5.1 µC cm^−2^. The ordering of the organic cations serves as the primary source of this spontaneous polarization.^[^ ^12^ ^]^ However, in the high‐temperature phase (Figure 1b), the space group transitions to the centrosymmetric *Cmca*, in which the CHA cations become disordered. This phenomenon resembles that observed in some recently discovered molecular ferroelectrics,^[^ ^10^, ^53^, ^54^ ^]^ where dynamic‐disordered organic cations at high temperatures become ordered structures at lower temperature. This transition results in symmetry‐breaking and the establishment of long‐range order with spontaneous electric polarization.

In this work, we adopted a low‐cost antisolvent method^[^ ^51^, ^55^ ^]^ with high repeatability to synthesize ferroelectric NSs at room temperature. As shown in Figure 1c, we rapidly injected the DMF precursor solution containing PbBr_2_ and (CHA)Br with a molar ratio of 1:2 into vigorously stirred toluene at room temperature. This rapidly reduces the solubility and leads to the immediate formation of the (CHA)_2_PbBr_4_ NSs. The formation of the NSs is indicated by the immediate color change of the solution, that is, from colorless to slightly turbid (Figure S1, Supporting Information).

Much research evidence has supported the application of organic ligands, such as octylammonium, to synthesize perovskite NSs so as to inhibit the further growth of NSs and improve their colloidal stability.^[^ ^56^, ^57^ ^]^ However, due to the 2D structure of (CHA)_2_PbBr_4_, these ligands may be incorporated into the perovskite structure and form NSs with an incorrect and non‐piezoelectric structure. As shown in Figure S2 (Supporting Information), the NSs synthesized with octylammonium do not match the X‐ray diffraction (XRD) simulations. These NSs do not exhibit piezoelectric responses (Figure S3, Supporting Information), which is inconsistent with our expectations. Therefore, organic ligands are not suitable for the synthesis of (CHA)_2_PbBr_4_ NSs in this work.

**Figure 2:**
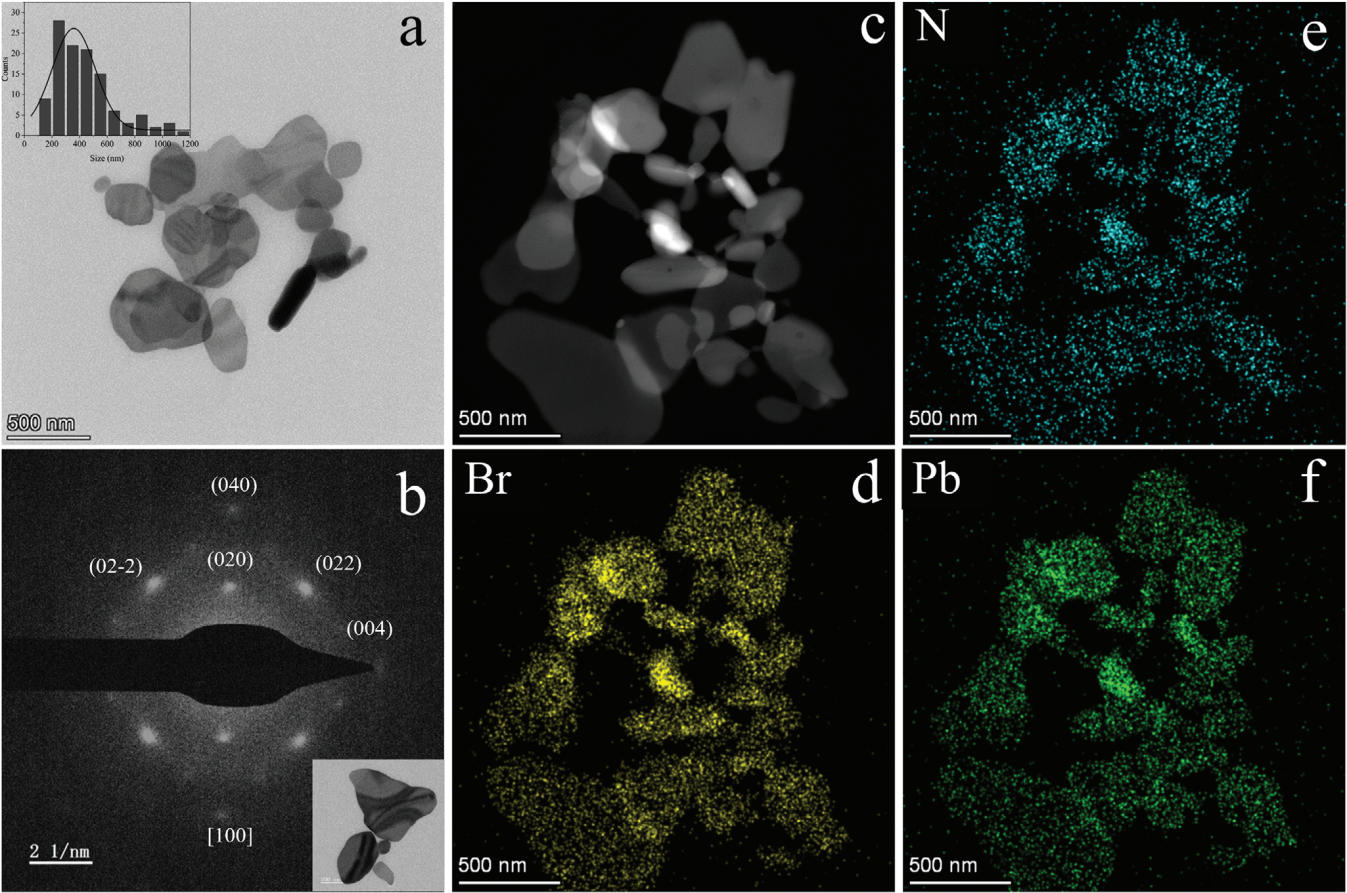
TEM images of (CHA)_2_PbBr_4_ NSs. a) TEM image and lateral size distribution of (CHA)_2_PbBr_4_ NSs. b) SAED pattern of a typical (CHA)_2_PbBr_4_ NS. Inset: corresponding TEM image. c‐f) STEM image and STEM‐EDS elemental mappings of (CHA)_2_PbBr_4_ NSs.

To investigate the structure and purity of the NSs, XRD was carried out on their drop‐casted films. Remarkably, the resultant diffraction peaks were consistent with the simulated patterns of bulk (CHA)_2_PbBr_4_ (Figure 1d), implying that the NSs also adopt a layered perovskite structure akin to the bulk crystal. Moreover, XRD images (Figure 1d) show sharp peaks, confirming the high crystallinity and purity of the NSs. The presence of periodic (h00) (h = 2, 4, 6, 8, 10, and 12) diffraction peaks denote the vertical stacking of the NSs. Specifically, the NSs with large lateral sizes (Figure 1e,f) lie flat on the substrate and overlap with each other. As a result, the NSs are stacked into superlattices, with preferred orientations along the (h00) direction, which is consistent with previous reports.^[^ ^50^ ^]^ Furthermore, we simulated the morphology of the single crystal (CHA)_2_PbBr_4_. As depicted in Figure S4 (Supporting Information), the predominantly exposed facet of the single crystal (CHA)_2_PbBr_4_ was identified as (200), corroborating the findings from selected area electron diffraction (SAED) patterns (**Figure** **2**b, indicating that the NSs are oriented toward the zone axis [100]) and XRD analyses. These results all indicate that the obtained hybrid perovskite NSs have with layered structure akin to the bulk crystal. Furthermore, we have established that the nanosheets possess good environmental and electrical stability (Figures S5 and S6, Supporting Information).

Interestingly, the collective findings from DSC and temperature‐dependent XRD studies indicate that the phase transition temperature of the nanosheets broadens and shifts toward lower temperatures (Figures S7–S9, Supporting Information), which is akin to the inorganic counterparts.^[^ ^58^, ^59^ ^]^ We summarized ferroelectricity‐related parameters of the nanosheets and some other OIHP ferroelectrics in Table S1 (Supporting Information).

Then atomic force microscopy (AFM) measurements were performed to investigate the morphology of the NSs. As shown in Figure S10 (Supporting Information), the NS thickness is in the range of 10 to 70 nm, which is suitable for the research of ferroelectric properties.

**Figure** **2**a displays typical transmission electron microscopy (TEM) images of the (CHA)_2_PbBr_4_ NSs. These NSs have lateral dimensions on the order of several hundred nanometers, with an average lateral size of 357.59 nm. Figure 2b presents the selected area electron diffraction (SAED) image obtained during TEM measurements, indicating the single‐crystalline nature of the NSs. The NSs are oriented toward the zone axis [100] and enclosed by (022), (020), (02‐2), (040), and (004) facets. The EDS spectrum in Figure S11 (Supporting Information) shows that the NSs contain C, N, Pb, and Br elements. In addition, the N, Pb, and Br elements are uniformly distributed, as shown in the elemental mapping (Figure 2c–f).

### 2.2 Observation of Ferroelectric Domains

**Figure 3:**
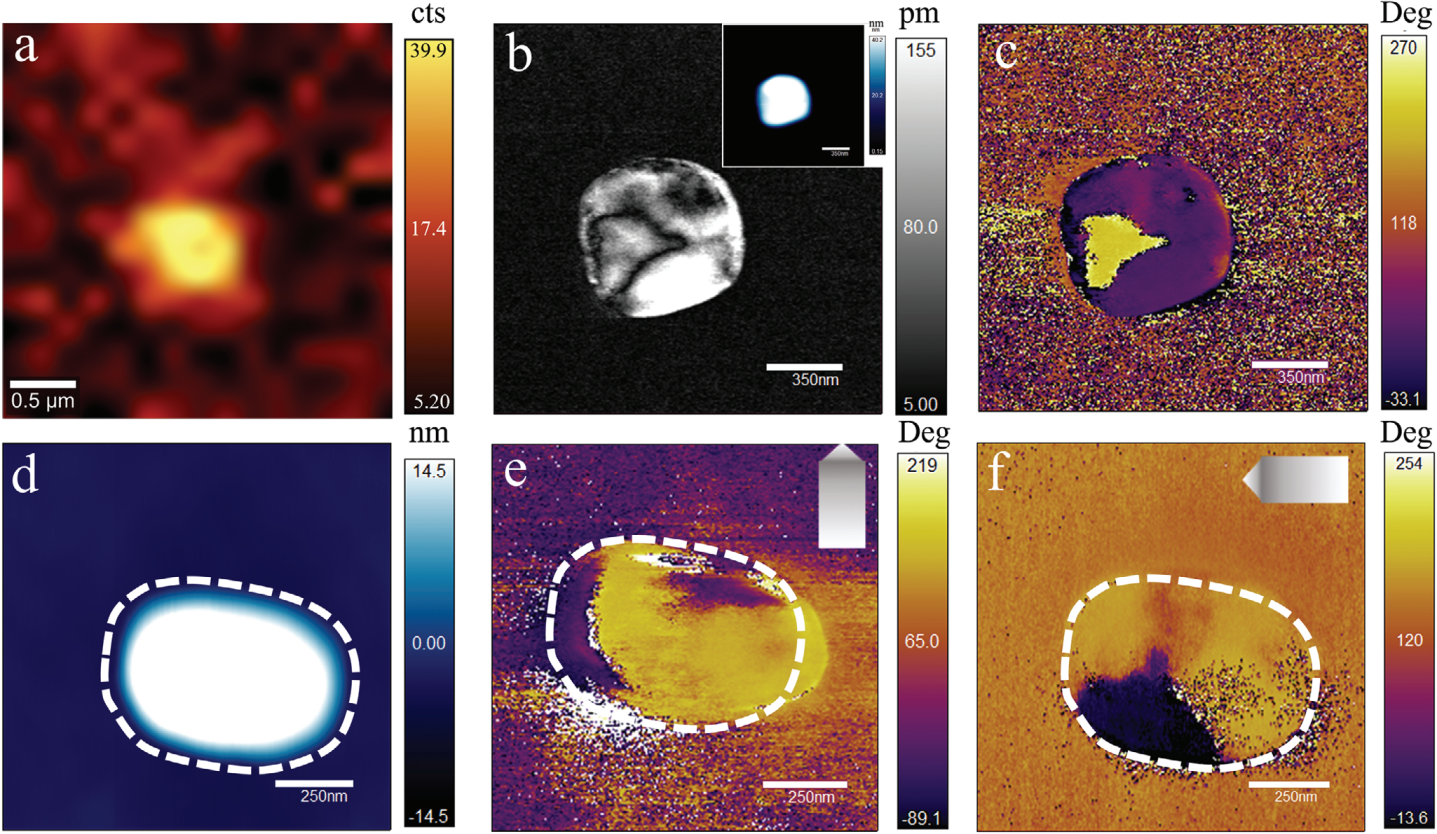
Observation of domains on (CHA)_2_PbBr_4_ NSs. a) SHG imaging of a single NS. Lateral PFM amplitude b) and phase c) of another NS. Inset: Corresponding topography image of the NS. Domain walls can be clearly observed in both amplitude and phase images. d) Topography image of another single NS. Lateral PFM phase of the NS in d) before e) and after f) a 90° clockwise rotation. The NS is marked by white dotted lines. The PFM cantilever is indicated by a grey arrow.

Second‐harmonic generation (SHG) microscopy is an effective probing tool that is sensitive to broken inversion symmetry and therefore serves as an excellent method to investigate ferroelectricity. Based on this, we first explored the structural symmetry through SHG scanning on a single NS at room temperature. The nonzero SHG (**Figure** **3**a) directly reveals the non‐centrosymmetric nature of the NS.

PFM measurements were conducted on the NSs to get further insight into their ferroelectricity. In principle, PFM detects the nanoscale domain structure by probing the deformation of a sample caused by the reverse piezoelectric effect through an electrical tip. Here, lateral PFM was used to probe the in‐plane polarization through the torsional motion induced by the nonzero shear piezoelectric effect. The PFM amplitude reflects the absolute magnitude of the local piezoelectric response, while the PFM phase indicates the polarization direction for individual domains. As shown in Figure 3b, compared with the highly doped Si substrate in the amplitude channel, the (CHA)_2_PbBr_4_ NS exhibited clear piezoelectric response and domain walls. Moreover, a 180° phase difference can be observed in the phase image (Figure 3c), corresponding to the two opposite polarization directions. These NSs tended to stay in the multi‐domain state at room temperature (more PFM observation results of domains in NSs can be found in Figure S12, Supporting Information). To prevent the morphology of the sample from affecting the PFM signal detection, we rotated the sample by 90° and detected the phase signal before and after the rotation. As shown in Figure 3e,f, the NS exhibited similar domain structures before and after the rotation, indicating that the phase signal is derived from real ferroelectric signals rather than artificial ones caused by the morphology of the sample.

### 2.3 Ferroelectric Switching

**Figure 4:**
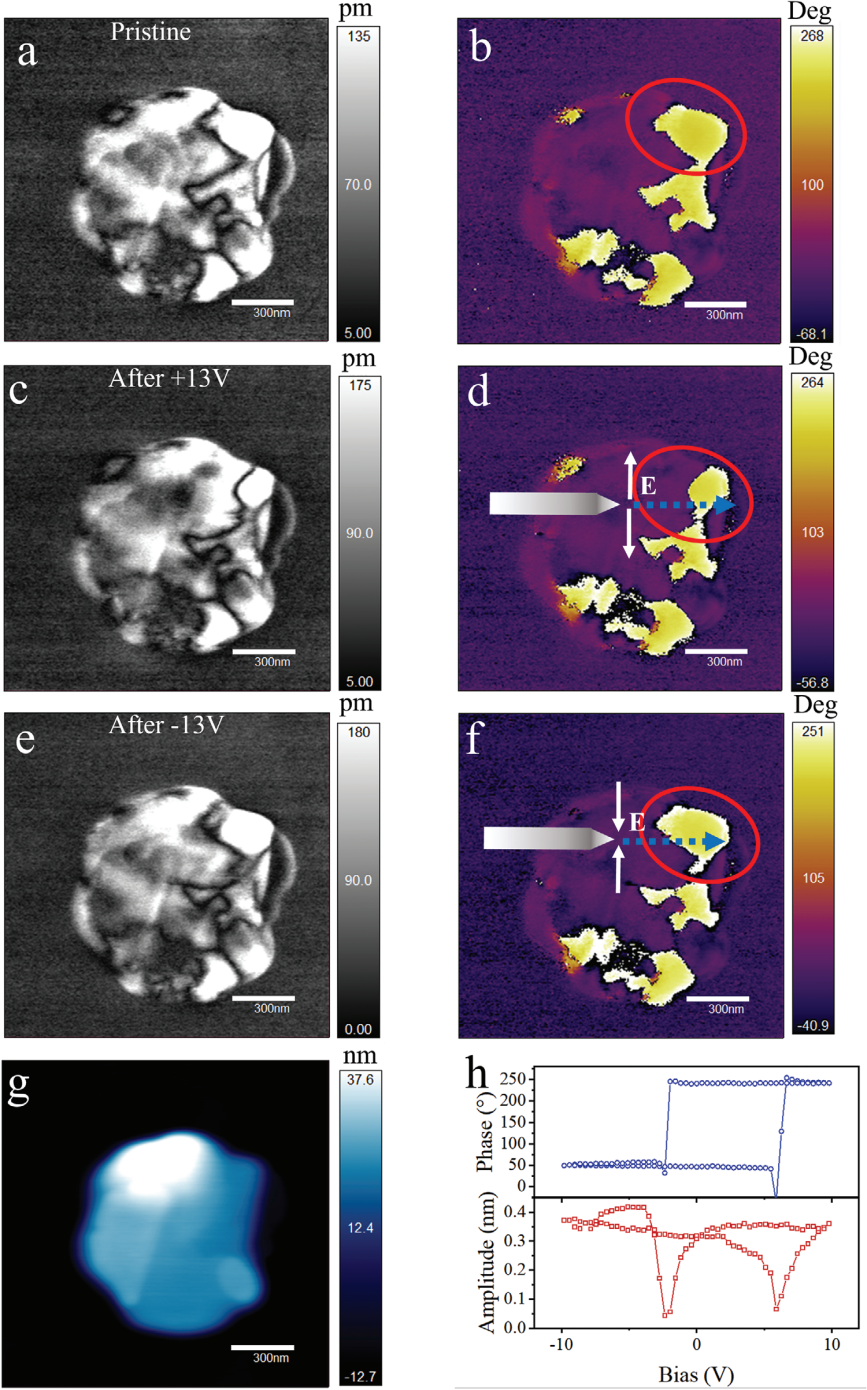
Ferroelectric polarization switching by PFM on (CHA)_2_PbBr_4_ NSs. The pristine state of the amplitude a) and phase b) of a single NS. PFM amplitude c) and phase d) images after moving the PFM tip along the blue dashed arrow with a tip bias of +13 V and a speed of 100 nm s^−1^. The region switched by the tip bias is marked by a red circle. PFM amplitude e) and phase f) images after moving the PFM tip along the red dashed arrow with a tip bias of −13 V and a speed of 100 nm s^−1^ for domain back‐switching. g) Surface topography image of the same NS. h) Off‐field local lateral PFM‐switching spectroscopy of the phase (top) and amplitude (bottom).

To investigate the domain switching behavior of (CHA)_2_PbBr_4_ NSs, we manipulated the spontaneous polarization by scanning the surface of a single NS with an electrically biased PFM tip. **Figure** **4**a,b shows the pristine domain patterns of the NS, in which domain walls appear as darker lines in the PFM amplitude image. Due to the in‐plane polarization, the NS exhibited almost no piezoelectric responses in the vertical PFM image (Figure S13, Supporting Information). By moving the electrically biased tip at +13 V along the blue dashed arrow with a speed of 100 nm s^−1^, the lateral component of the tip electric field reversed the in‐plane polarization of the domain. The color of the domain area marked by the red circle changed from yellow to purple (Figure 4d). By further moving the electrically biased tip along the blue dashed arrow at −13 V with a speed of 100 nm s^−1^, the color of the domain returned to yellow (Figure 4f). This verifies the switching behavior of the domain. In another switching experiment, the ferroelectric domains after switching can remain stable for 12 h (Figure S14, Supporting Information), which excludes the possibility that the phase signals are derived from artificial signals generated by charge injection. Another method is PFM switching spectroscopy. At a selected position, when the triangular pulse voltage applied by the tip exceeds the coercive electric field of the NS, the phase exhibited a clear 180° reversal, and the amplitude was at a minimum value, resulting in a butterfly‐shaped curve (Figure 4h). Moreover, we obtained a ferroelectric hysteresis loop from drop‐casted films of NSs on interdigitated electrodes (Figures S15 and S16, Supporting Information). As a result, the polarization reversibility of (CHA)_2_PbBr_4_ NSs is demonstrated, indicating the robust ferroelectricity of the NSs.

## 3 Conclusion

In summary, we adopted a room‐temperature antisolvent method to synthesize free‐standing 2D OIHP ferroelectric NSs with high repeatability and low cost. After that, XRD and TEM measurements were conducted to confirm the crystallinity and purity of the ferroelectric NSs. With PFM, ferroelectric domains in the NSs were clearly visualized and easily manipulated. The domain switching and PFM‐switching spectroscopy indicate the robust ferroelectricity of the (CHA)_2_PbBr_4_ NSs. This work demonstrates a low‐cost and scalable method for preparing ferroelectric nanomaterials, which promotes the research and applications of ferroelectric nanomaterials.

## 4 Experimental Section

### Materials

Lead bromide (PbBr_2_, 98%) was purchased from Aladdin. N, N‐dimethyl methanamide (DMF, 99.8%) was purchased from Macklin. Toluene (99.8%), HBr aqueous solution (40%), cyclohexylamine (99%), and ethanol (99.7%) were purchased from Sinopharm. All the chemicals were used as received without further purification.

### Synthesis of Cyclohexylammonium Bromide ((CHA)Br)

Cyclohexylamine (0.1 mol) was added to the ethanol in a single‐neck round bottom flask. A water bath was placed around this flask. HBr (0.11 mol) was then added dropwise to the stirring solution using a dropping funnel. The reaction was allowed to proceed for 2 h at room temperature under ambient conditions. The volatiles were then removed from the products using a rotary evaporator, leaving behind the solids. The solids were washed several times with diethyl ether.

### Synthesis of 2D (CHA)_2_PbBr_4_ Nanosheets (NSs)

In a typical synthesis of (CHA)_2_PbBr_4_ NSs, 0.2 mmol of (CHA)Br and 0.1 mmol of PbBr_2_ were dissolved in 2 mL of DMF to form a perovskite precursor solution at room temperature. Then, 15 µL of the perovskite precursor solution was quickly dropped into 10 mL of toluene under vigorous stirring. The NSs formed immediately, as the solution got turbid. The (CHA)_2_PbBr_4_ NSs were obtained after centrifugation at 7,000 rpm for 1 min. Finally, these NSs were redispersed in toluene for further characterization.

### Material Characterization

The X‐ray diffraction (XRD) patterns were recorded by a Rigaku SmartLab SE X‐ray diffractometer with Cu Kα radiation (λ = 1.5406Å, 40 kV, 40 mA). Samples used for Transmission electron microscopy (TEM), XRD, and piezoresponse force microscopy (PFM) characterizations were prepared by dropping a colloidal dispersion in toluene onto the amorphous carbon‐coated copper grids, glass, and highly doped silicon, respectively, and then dried at 333K. TEM and energy‐dispersive X‐ray spectroscopy (EDS) mapping images were recorded on Talos F200X. Differential scanning calorimetry (DSC) experiments were performed on a NETZSCH DSC 200 F3 instrument under a nitrogen atmosphere in aluminum crucibles with a heating and cooling rate of 10 K min^−1^.

### SHG Imaging

SHG imaging was measured using a home‐built optical path coupling with a commercial confocal scanning microscope (Witec alpha R300). The input laser was provided via Coherent Lasers (Chameleon Ultra II), with 80 MHz repetition frequency and 150 femtosecond pulse width. A 100X objective (N.A = 0.9, Zeiss) was selected to focus the laser onto the nanosheets and collect the reflected signals. A dichroic mirror (DMSP650R, Thorlabs) was used to reflect the input laser onto the sample and then pass through the generated SHG signal reflected from the same site.

### AFM and PFM Measurements

The topographic images of the as‐prepared NSs were measured by AFM at contact mode (Asylum Research MFP‐3D). The ferroelectric domain structures of these nanodots were characterized by PFM (Asylum Research MFP‐3D) using conductive probes (EFM, Nanosensor). The local piezoresponse loop measurements were carried out by fixing the PFM probe on a selected NS and then applying a triangle‐square waveform accompanied by an AC‐driven voltage via the conductive PFM probe. To improve the PFM sensitivity, a dual‐frequency resonance‐tracking technique was adopted, which was also provided by Asylum Research.

## Conflict of Interest

The authors declare no conflict of interest.

## Supporting information

Supporting Information

## Data Availability

The data that support the findings of this study are available in the supplementary material of this article.
