# Supplementary material for: Organic–Inorganic Hybrid Perovskite Ferroelectric Nanosheets Synthesized by a Room‐Temperature Antisolvent Method
Source: Adv Sci (Weinh). 2024 May 22;11(29):2400636. doi: 10.1002/advs.202400636 (PMC11304249; doi:10.1002/advs.202400636)
Supplement: Supplementary file 1 — Supporting Information [file ADVS-11-2400636-s001.docx]

Supporting Information

**Organic–inorganic hybrid perovskite ferroelectric nanosheets synthesized by a room-temperature antisolvent method**

*Tai-Ting Sha ^†[a]^, Xing-Chen Zhang^†[b]^, Ru-Jie Zhou^†[a]^, Guo-Wei Du^[a]^, Yu-An Xiong^[a]^, Qiang Pan^[a]^, Jie Yao^[a]^, Zi-Jie Feng^[a]^, Xing-Sen Gao*^[b]^, and Yu-Meng You*^[a]^*


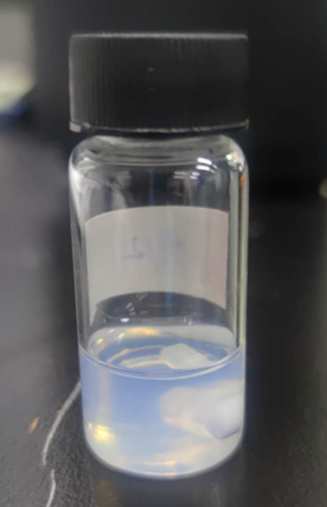

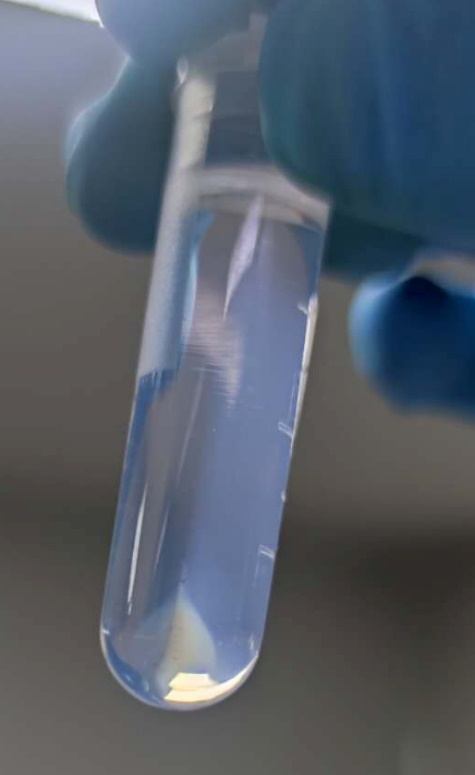


**Figure S1.** Photographs of (CHA)_2_PbBr_4_ NSs solutions and after the centrifugation


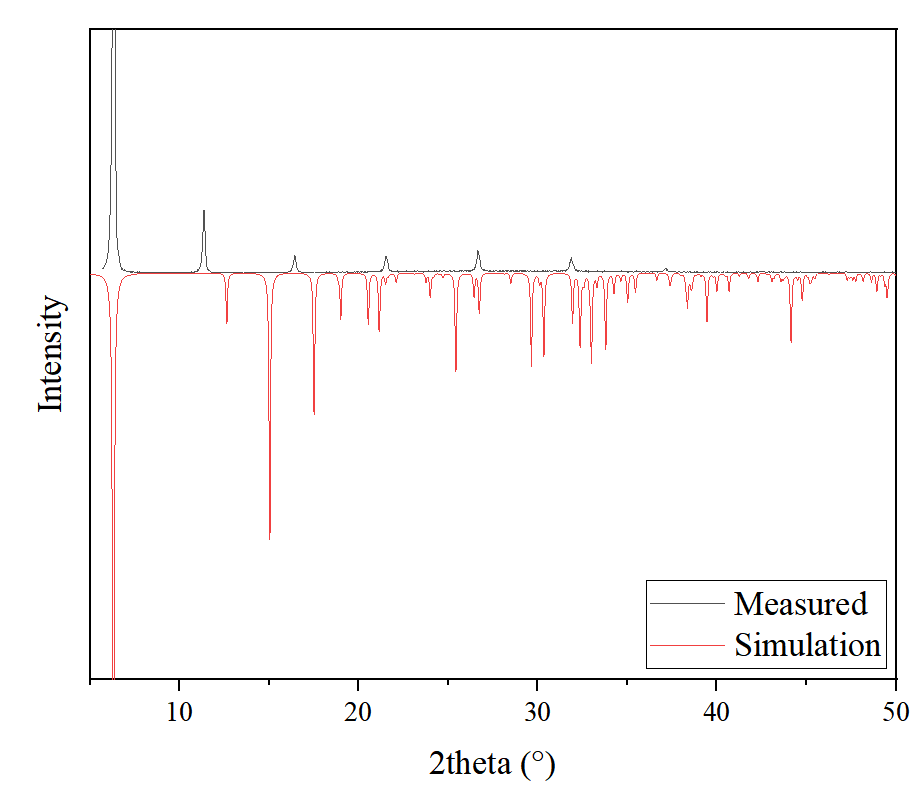


**Figure S2.** XRD patten of NSs synthesized with the addition of octylammonium, which does not match the simulation.


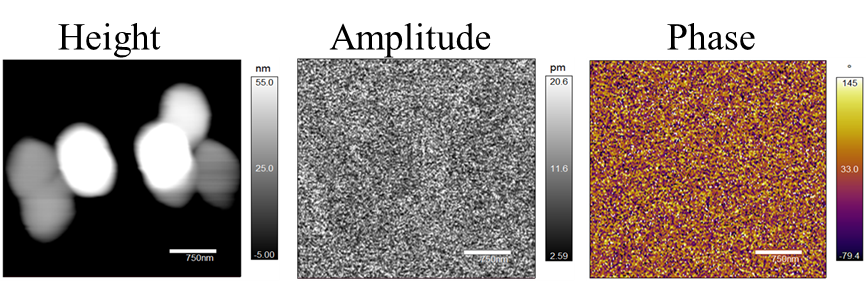


Figure S3. PFM amplitude and phase of NSs synthesized with oleyl amine. These NSs exhibit no piezoelectric response.


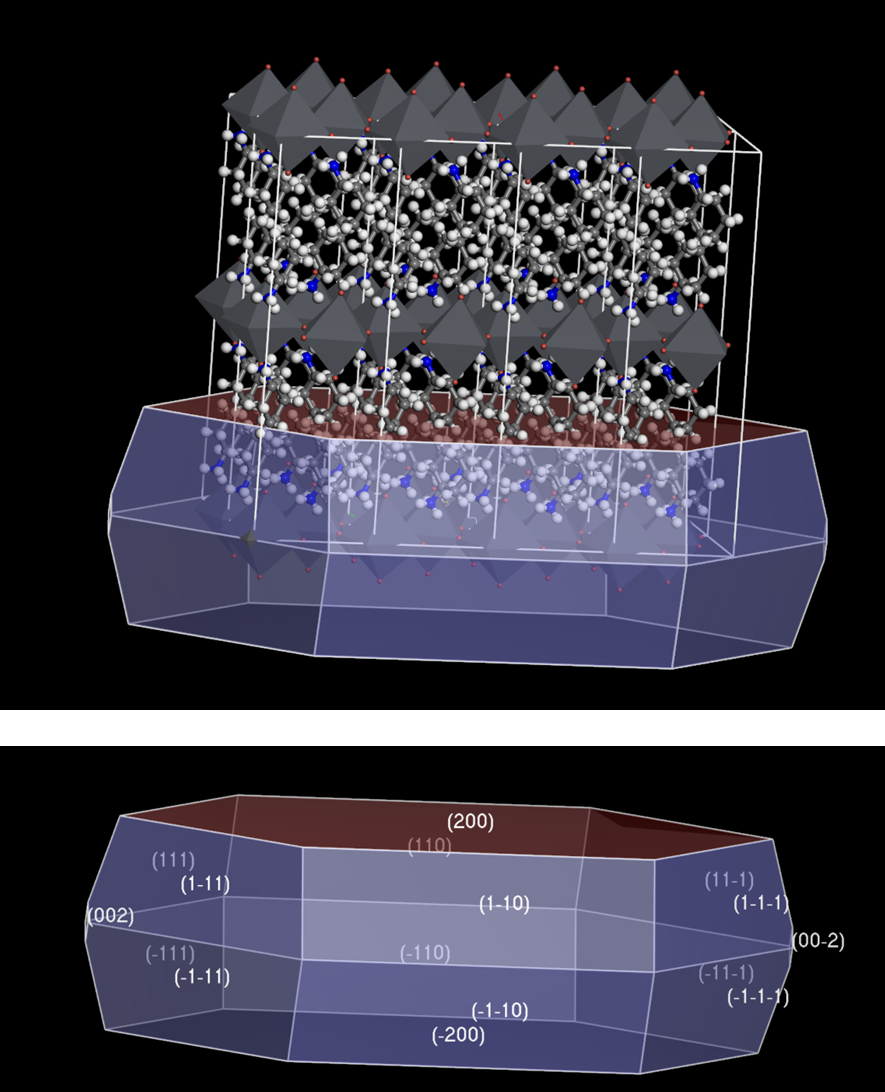


**Figure S4.** Simulated morphology of the single crystal (CHA)_2_PbBr_4_. The largest exposed facet (200) is colored by red.

Compared with 3D perovskites, the 2D perovskites have a larger formation energy, a higher moisture stability, and a longer lifetime^[1-2]^. By the reason of their 2D perovskite structure, the NSs exhibit a much better stability than their 3D perovskite counterparts. Firstly, regarding environmental stability, we conducted PXRD and SHG intensity tests on drop-casted film of NSs before and after being stored under ambient conditions (relative humidity of approximately 50%) for 8 days. Following the 8-day duration, the SHG intensity of NSs only slightly weakened (**Figure S5b**). The NSs experienced only a minor reduction in crystallinity, as evidenced by the slight decrease in PXRD intensity (see **Figure S5c**). The overall findings indicated the good stability of NSs under environmental conditions.

Additionally, to evaluate the electrical stability of NSs, we performed PFM switching spectroscopy on a single NS with a thickness of 20 nm for 100 times. No obvious degradation was observed in the local lateral PFM-switching spectroscopies over 100 cycles, indicating good electrical stability of NSs (**Figure S6**).


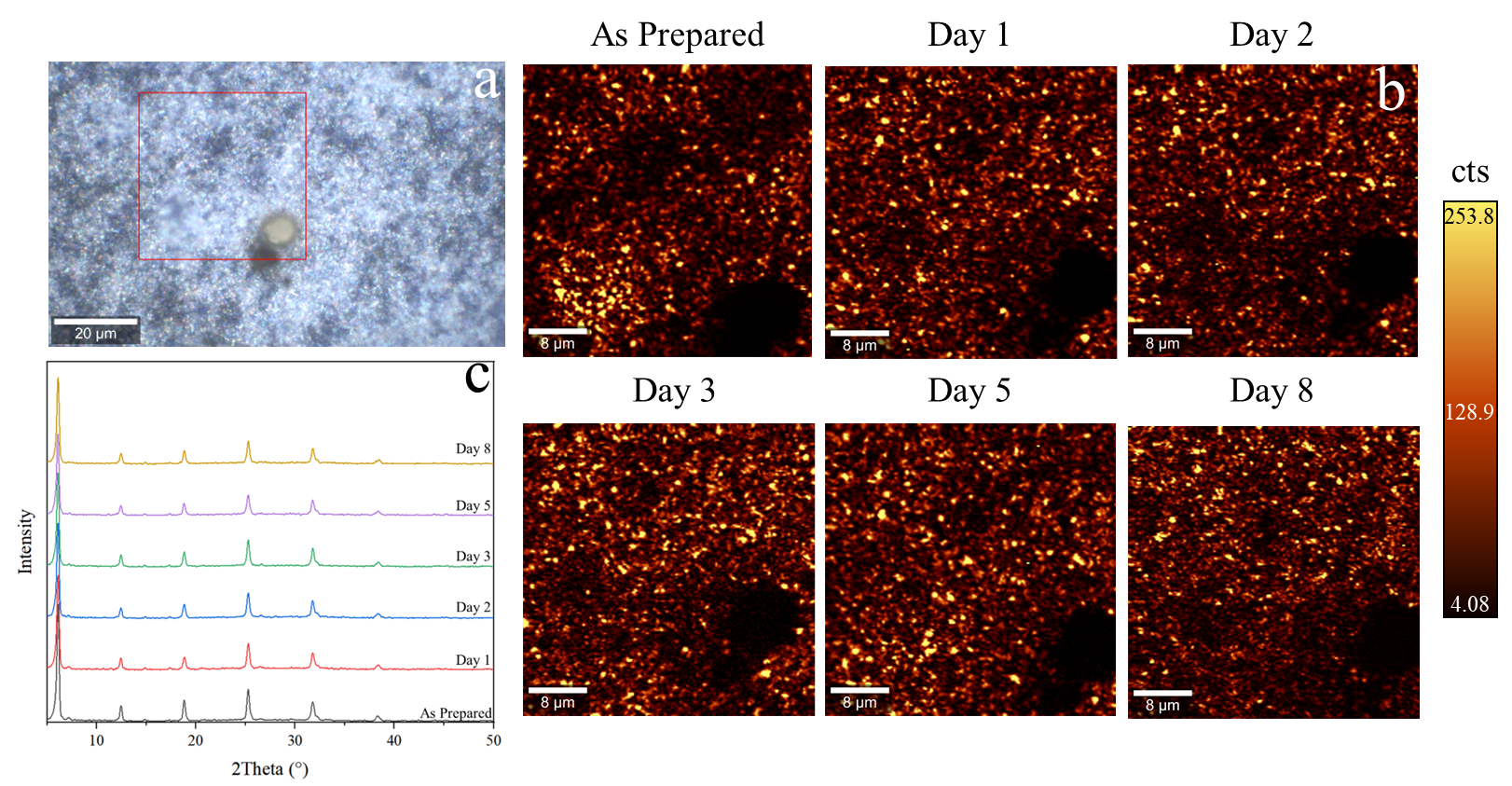


**Figure S5.** Environmental stability of NSs. a) Optical microscopy image of the drop-casted film of NSs. The region for SHG imaging is marked by a red box. b) SHG imaging of the red box region after being stored at ambient conditions for 8 days. c) PXRD patten of the drop-casted film of NSs after being stored at ambient conditions for 8 days.


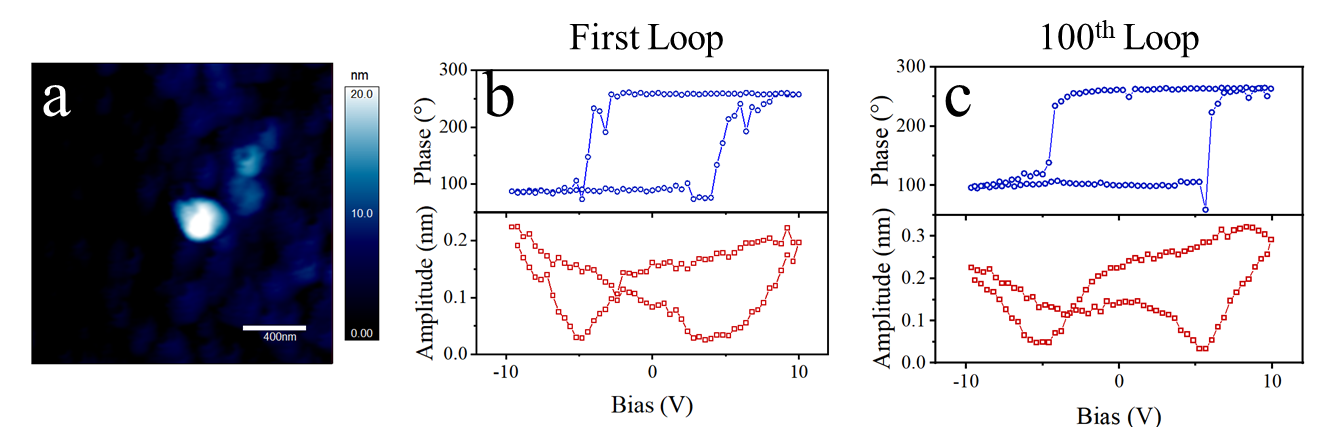


**Figure S6.** Electrical stability of NSs. a) Surface topography image of a single NS with a thickness of 20 nm, where PFM switching spectroscopy was performed. Off-field local lateral PFM-switching spectroscopy of the phase and amplitude for the first b) and 100^th^ c) loop.

Typically, a ferroelectric tends to experience a structural phase transition from a centrosymmetric high temperature phase to a polar low temperature phase. To determine the phase transition temperature of the NSs, we performed differential scanning calorimetry (DSC) performed in the range of 330-380 K. And the *T*_c_ was identified with the temperature of the peak in the heat flow. The *T*_c_ was found to shift from 365 K in bulk to 361 K in NSs, meanwhile there was also an increase in the peak width in the NSs (Figure S7a and 7c). Furthermore, temperature dependent XRD measurements of both the NSs and bulk crystal powder were performed in a temperature range from 320 to 385K. And cell parameter a for bulk and NSs were calculated from temperature dependent XRD patterns. As shown in Figure S7b, there is an abrupt shift in cell parameter a at 365 K, that is transition temperature. In contrast to the behavior of the bulk material, the NSs undergo a more gradual change in cell parameter a from 348 to 361 K (Figure S7d). And the cell parameter a of NSs (28.7997(7), 378K) is slightly smaller than that in bulk (29.0250(4), 378K), which is akin to the inorganic perovskites. The findings from DSC and XRD studies collectively suggest that the phase transition temperature of NSs broadens and shifts towards lower temperatures. This phenomenon resembles that of inorganic ferroelectrics^[3-4]^. This result implies that these hybrid perovskite NSs may demonstrate a comparable size-effect to their inorganic counterparts, despite differing mechanisms of ferroelectricity (displacement-type for inorganic perovskite ferroelectrics and order-disorder-type for hybrid perovskite ferroelectrics).


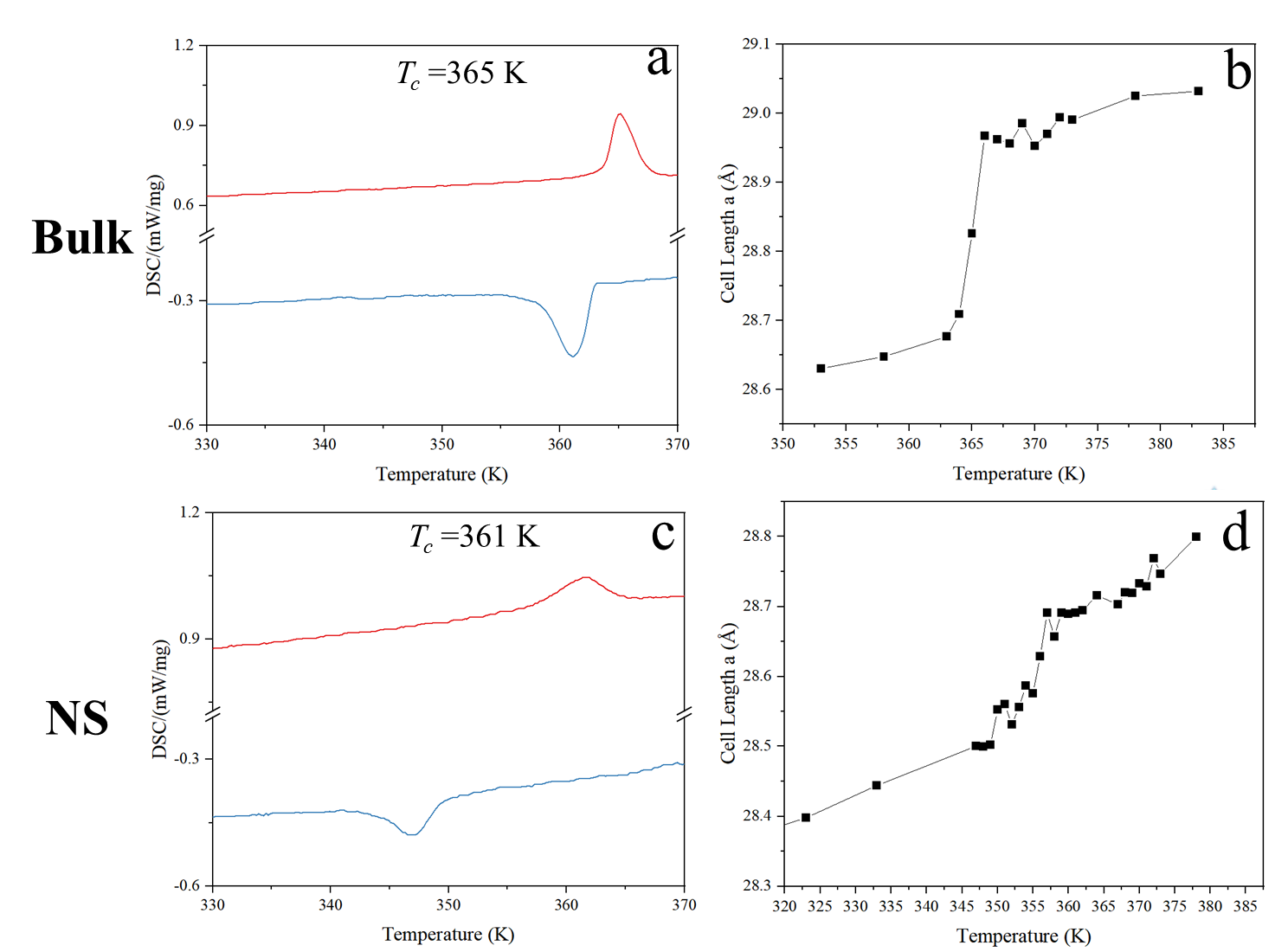


Figure S7. Differential scanning calorimetry (DSC) for a) bulk, b) NSs. Change in cell parameter a for b) bulk, d) NSs calculated from temperature dependent XRD patterns.


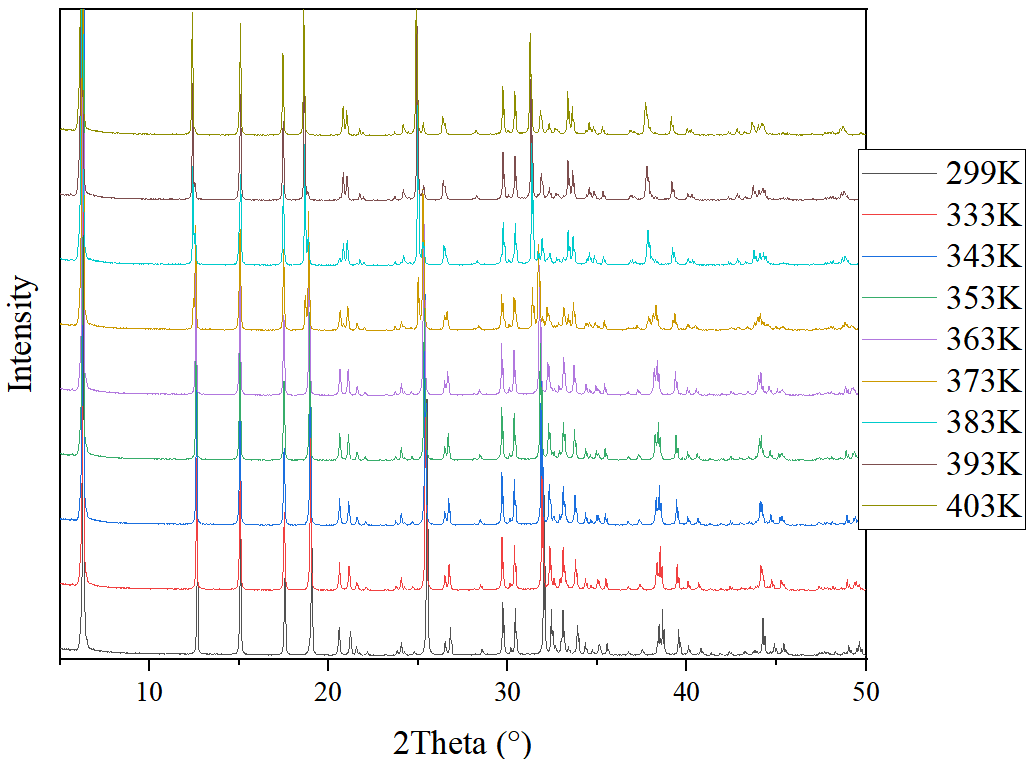


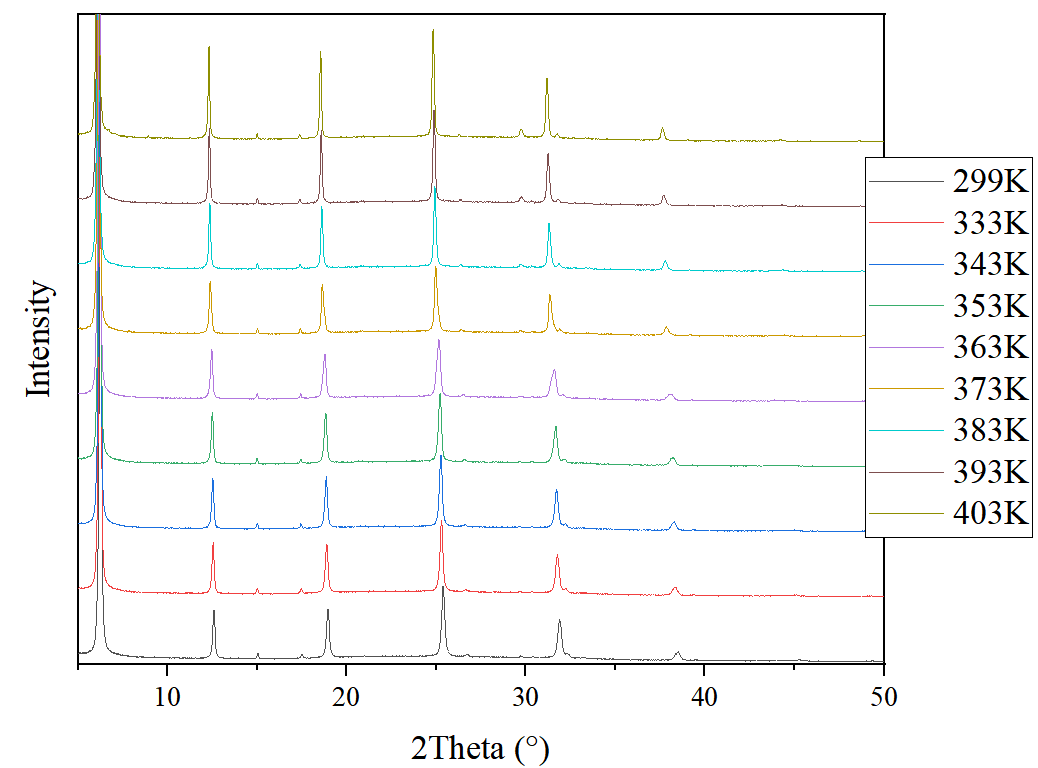


**Figure S8.** Temperature dependent XRD patten of bulk (top) and NSs (bottom)


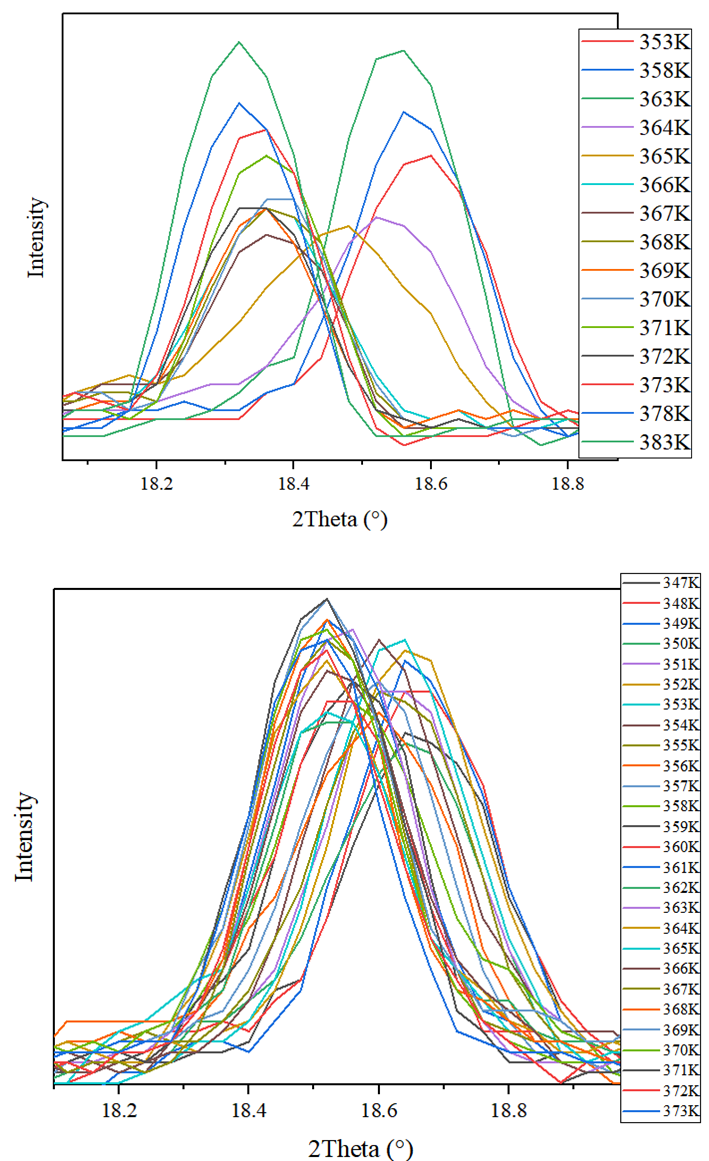


**Figure S9.** Temperature dependent XRD patten of bulk (top) and NSs (bottom).

Compared with 3D organic–inorganic hybrid perovskites (OIHPs), 2D OIHPs have a larger formation energy, a higher moisture stability, and a longer lifetime^[1-2]^. Moreover, their 2D perovskite structures provide more diversity and higher tunability of structural and physical characteristics. Although there are plenty of reports on the synthesis of new 2D OIHP ferroelectrics, OIHP ferroelectric nanomaterials are rarely reported. And it is challenging to obtain the ferroelectricity-related parameters of these OIHP nanomaterials, particularly *P*_s_. Noticeably, Wang et. al.^[5]^ studied the in-plane ferroelectric properties in mechanical exfoliated BA_2_PbCl_4_ thin flakes，in which the ordering of electric dipoles could be preserved down to one unit-cell thickness or two vdW layers at room temperature. But phase transition behaviors and hysteresis loops were not involved in their work.

We endeavored to study the phase transition behaviors and hysteresis loops of (CHA)_2_PbBr_4_ NSs and summarized the ferroelectric performance of halide perovskites with similar structures in Table S1. As shown in the table, most lead OIHP ferroelectrics crystallize in the polar space group *Cmc*2_1_ (point group *mm*2) in the ferroelectric phase, while the space group transitions to the centrosymmetric *Cmca, Cmcm* or *Pbca* (they all belong to the point group *mmm*) in the paraelectric phase. These ferroelectric-paraelectric transitions can be described as the Aizu notation of *mmm*F*mm*2. From ferroelectric phase to paraelectric phase, symmetry elements reduce from 8 (point group *mm*2) to 4 (point group *mmm*). Thus, these 2D lead OIHP ferroelectrics are uniaxial ferroelectrics with two equivalent polarization directions. Among these 2D OIHP ferroelectrics, (CHA)_2_PbBr_4_ possess favorable *T*_c_ and *P*_s_, and the *T*_c_ of (CHA)_2_PbBr_4_ NSs is only 4 K lower than that of bulk crystal. These features make (CHA)_2_PbBr_4_ NSs a potential candidate for applications in high-density memory devices and piezoelectric catalysis.

| **Compounds** | **Ferroelectric Phase** | **Paraelectric Phase** | **Aizu Notation** | ***T*_c_ (K)** | ***P*_s_**  **(μC cm^-2^ )** | **Number of**  **ferroelectric**  **axes** | **Ref** |
| --- | --- | --- | --- | --- | --- | --- | --- |
| (CHA)_2_PbBr_4_ nanosheets | *Cmc2*_1_ | *Cmca* | *mmm*F*mm*2 | 361 | - | 1 | This Work |
| Bulk (CHA)_2_PbBr_4_ | *Cmc2*_1_ | *Cmca* | *mmm*F*mm*2 | 365 | 5.8 | 1 | [6-7] |
| Mechanical exfoliated  BA_2_PbCl_4_ thin flakes | *Cmc2*_1_ | - | - | - | - | - | [8] |
| Bulk BA_2_PbCl_4_ | *Cmc2*_1_ | *I*4*/mmm* | 4/*mmm*F*mm*2 | 438 | 13 | 2 | [9] |
| [(CH_3_)_2_CHCH_2_NH_3_]_2_PbCl_4_ | *Pm* | *Pnma* | *mmm*F*m* | 302 | 4.8 | 2 | [10] |
| (3,3-DFCBA)_2_CuCl_4_ | *Cc* | *P*4_2_/*mmc* | 4/mmmFm | 380 | 0.29 | 4 | [11] |
| (4,4-DFCHA)_2_PbI_4_ | *Cmc2*_1_ | *Pbca* | *mmm*F*mm*2 | 377 | 4.5 | 1 | [12] |
| (BPA)_2_PbBr_4_ | *Cmc2*_1_ | *I*4/*mmm* | 4/*mmm*F*mm*2 | 375 | 4.8 | 2 | [13] |
| (CH_3_OC_3_H9N)_2_CsPb_2_Br_7_ | *Cmc2*_1_ | *Cmcm* | *mmm*F*mm*2 | 370 | 4.1 | 1 | [14] |
| (BA)_2_(FA)Pb_2_Br_7_ | *Cmc2*_1_ | *Cmcm* | *mmm*F*mm*2 | 322 | 3.8 | 1 | [15] |

**Table S1.** Ferroelectricity-related parameters of the (CHA)_2_PbBr_4_ nanosheets and other halide perovskite ferroelectrics with similar structures.


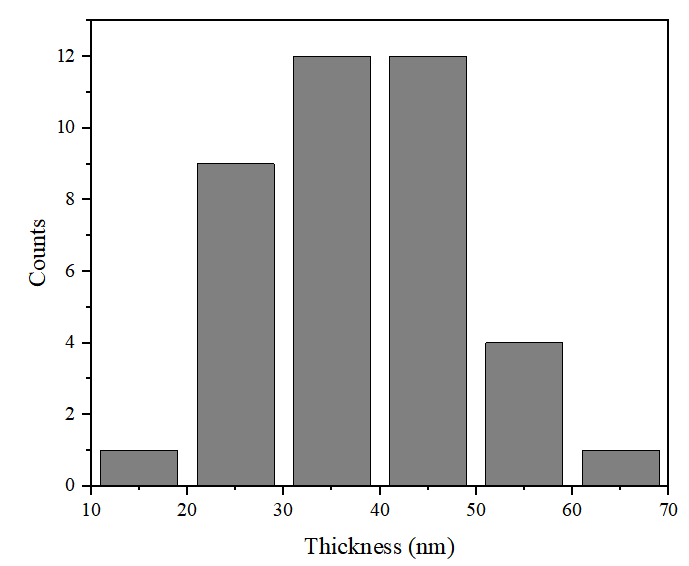


**Figure S10.** Thickness distribution plot of NSs. The thickness distribution of NSs was obtained by conducting AFM on a drop-casted film covering a 90×90 μm range. The heights of NSs were determined from the original AFM height profiles, and a thickness distribution plot was created.


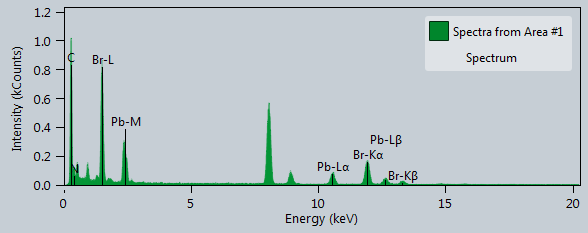


**Figure S11.** EDS spectra of (CHA)_2_PbBr_4_ NSs


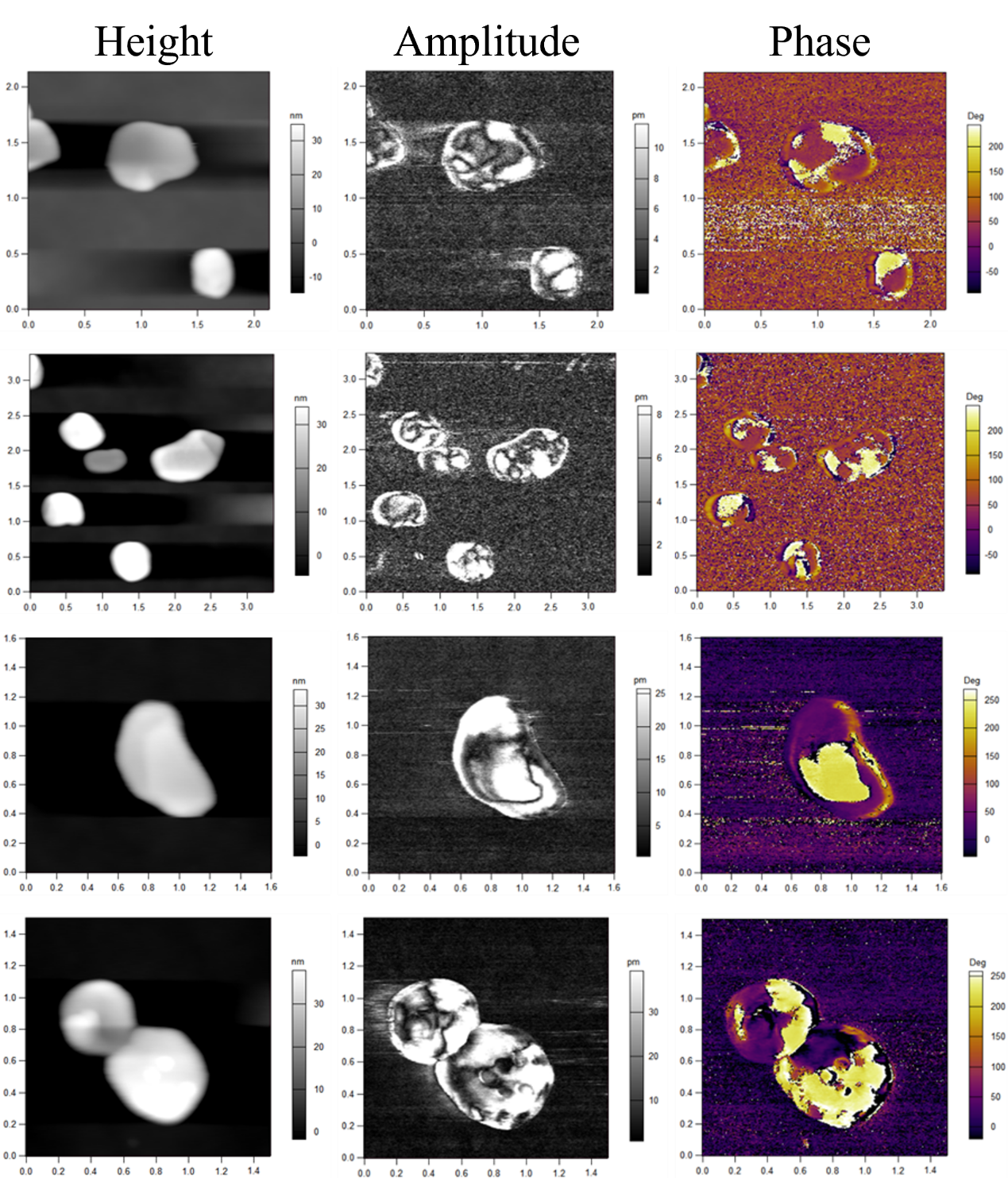


**Figure S12.** Domains observed in (CHA)_2_PbBr_4_ NSs.


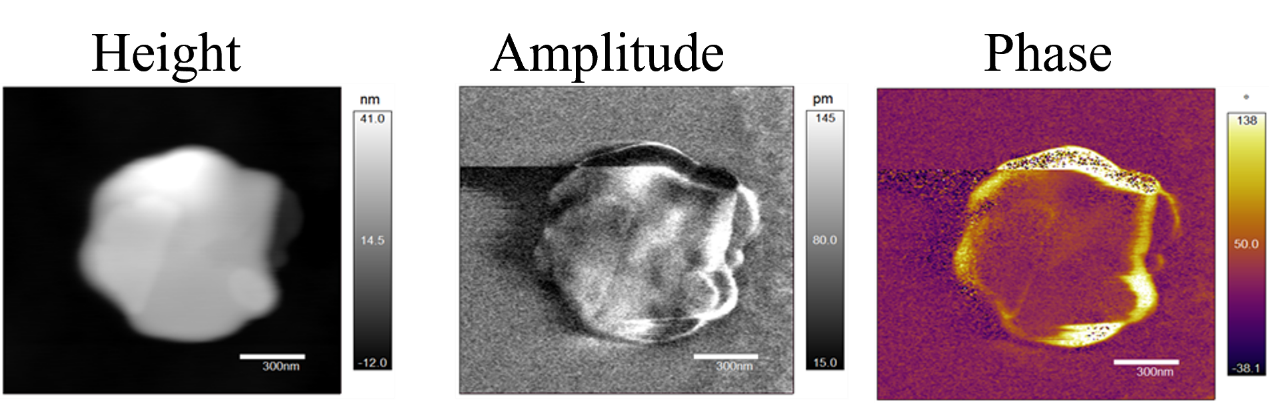


**Figure S13.** Vertical PFM of the NS in figure 4, which exhibit no ferroelectric response in the out of plane direction.


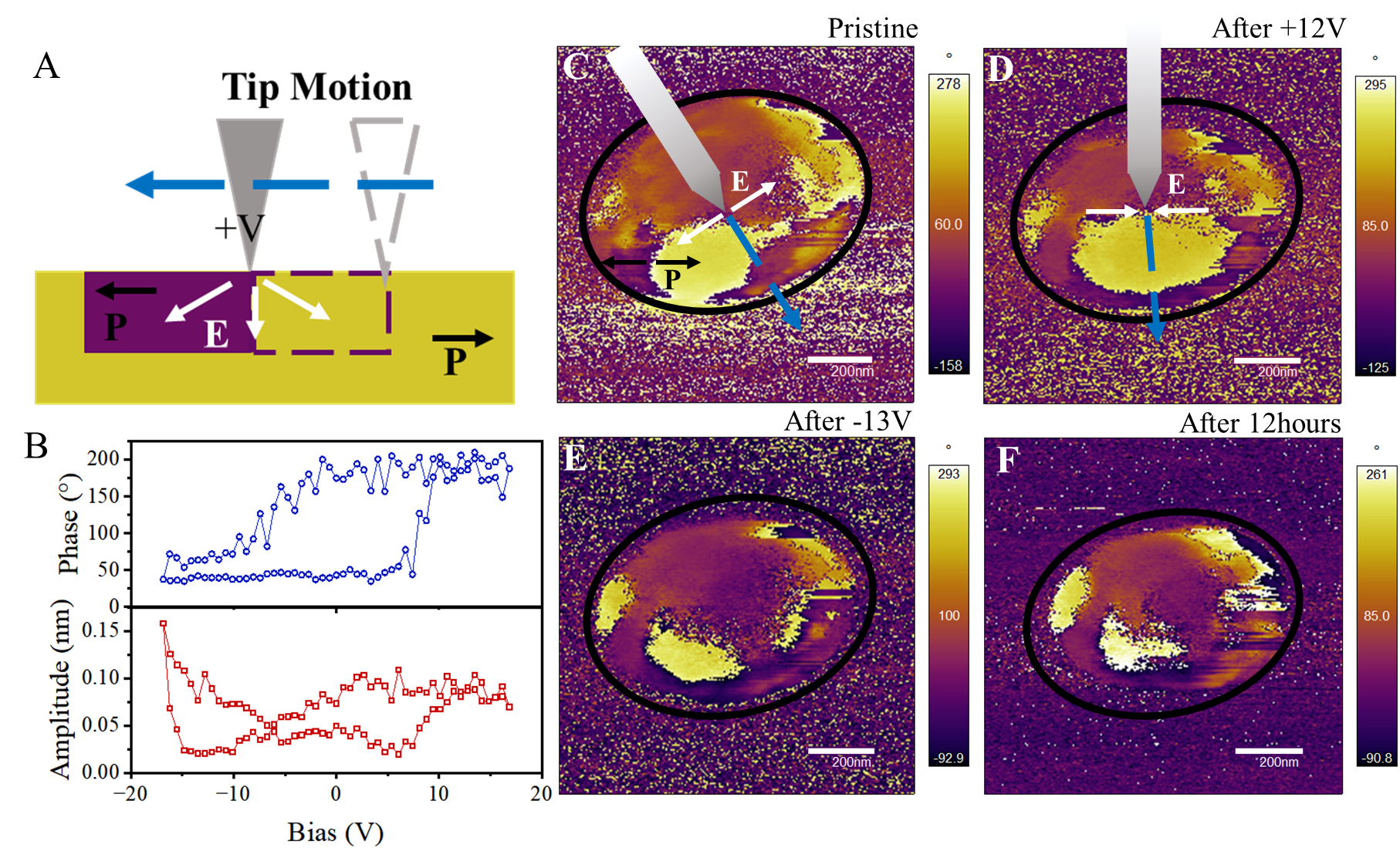


**Figure S14**. Ferroelectric polarization switching by PFM on (CHA)_2_PbBr_4_ NSs (A) Schematic showing the domain switched by the lateral component of the tip field. (B) Local lateral PFM switching spectroscopy of a single (CHA)_2_PbBr_4_ NS. (C) Lateral PFM phase image of the initial state of a single (CHA)_2_PbBr_4_ NS. The NS is marked by a black circle, and the polarization directions of the domains are indicated by the black arrows. The polarization switching was performed by moving the PFM tip along the blue line with the tip bias of +12 V at a speed of 1 μm s^-1^. (D) Lateral PFM phase image of the NS after the first domain switching, and the domain on the right side of the tip moving path changes from purple to yellow. And a back-switching operation was performed by moving the PFM tip along the blue arrow with the tip bias of -13 V at a speed of 1 μm s^-1^. (E) Lateral PFM phase image of the NS after the succeeding back-switching operation, and the domain on the right side of the tip moving path changes from yellow to purple. (F) Lateral PFM phase image of the NS obtained after 12 hours.

Polarization vs electric field loop (*P*-*E* loop) is the most convincing evidence for ferroelectricity. In practice, polarization is usually obtained by measuring the surface charge *Q* induced by polarization under an external voltage *V*, and the polarization *P* is determined by dividing the surface charge *Q* by the electrode area *S*. The electric field *E*, the applied voltage *V* is divided by the sample thickness *d*.


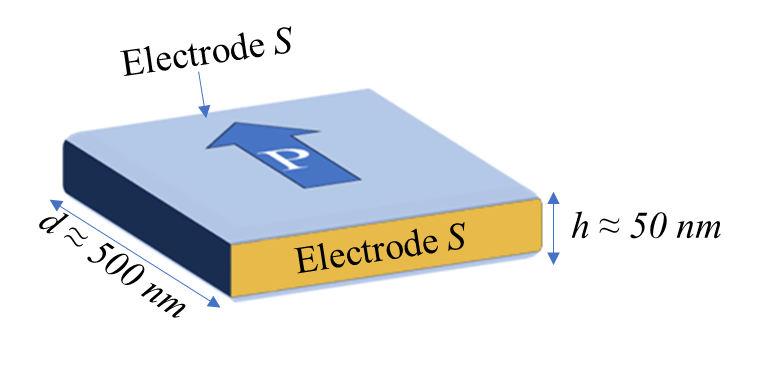


**Figure S15**. Schematic diagram of an ideal experimental layout for obtain a quantitative *P*-*E* loop on a single NS.

However, conducting measurements of *P*-*E* loops on nanomaterials presents significant challenges, particularly regarding in-plane polarization. Despite extensive research on ferroelectric nanomaterials, qualitative hysteresis loops are rarely reported. Most articles, even those from renowned journals, depend on PFM hysteresis loops as evidence of ferroelectricity^[8, 16-18]^. As mentioned above, qualitative hysteresis loops require a pair of electrodes perpendicular to the polar axis, which is difficult to achieve due to the lateral nanoscale dimensions of the single NS (Figure S15). Additionally, the surface charge *Q* resulting from polarization reversal is often too small to be detected accurately. Despite these obstacles, we endeavored to measure the *Q*−*V* curve on NSs. We obtained a ferroelectric hysteresis loop from drop-casted films of NSs on interdigitated electrodes, with a spacing of 3 μm, as shown in Figure S16a. The obtained *Q*−*V* curve is shown in Figure S16b, which may be resulted from multiple randomly stacked NSs between electrodes and values of *P* and *E* are hardly estimated.


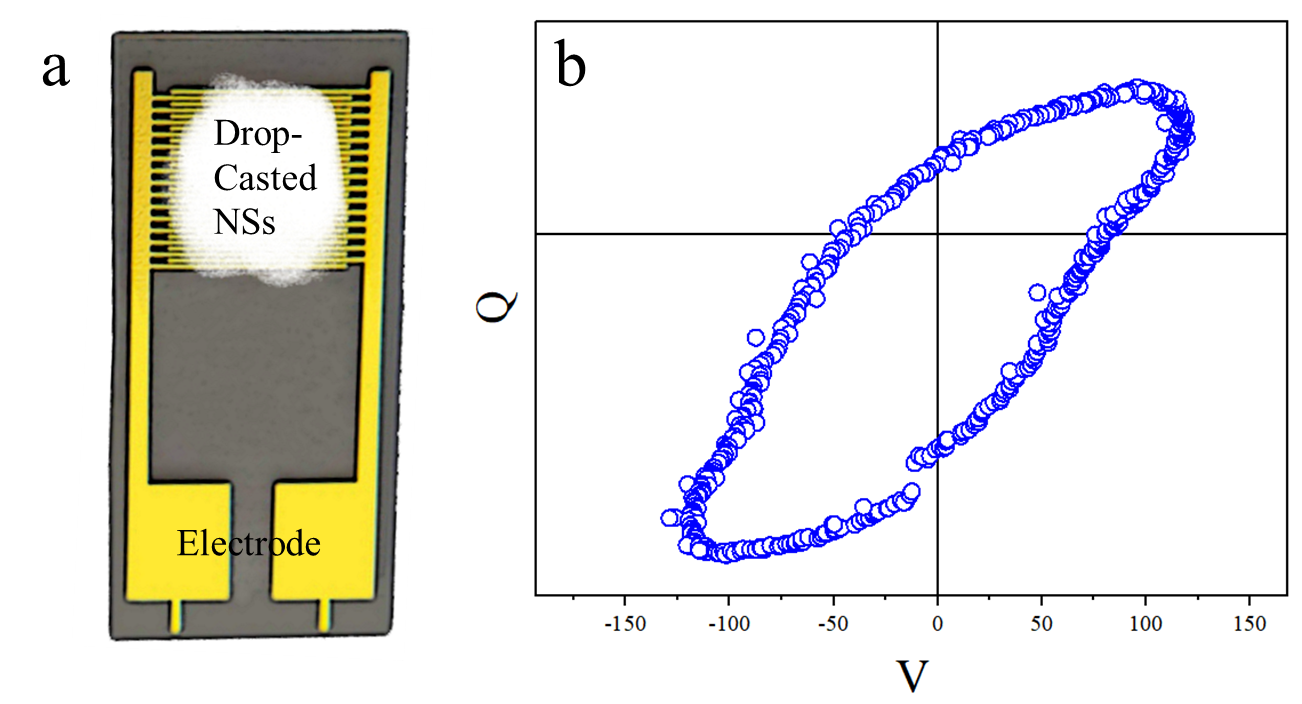


**Figure S16**. a) Schematic diagram of the drop-casted film of NSs on interdigitated electrodes. b) Ferroelectric hysteresis loop measured on drop-casted films of NSs on interdigitated electrodes.

[1] H. Tsai, W. Nie, J.-C. Blancon, C. C. Stoumpos, R. Asadpour, B. Harutyunyan, A. J. Neukirch, R. Verduzco, J. J. Crochet, S. Tretiak, *Nature* **2016**, *536* (7616), 312.

[2] Z. Wang, Q. Lin, F. P. Chmiel, N. Sakai, L. M. Herz, H. J. Snaith, *Nature Energy* **2017**, *2* (9), 1.

[3] S. Chattopadhyay, P. Ayyub, V. Palkar, M. Multani, *Physical review B* **1995**, *52* (18), 13177.

[4] M. B. Smith, K. Page, T. Siegrist, P. L. Redmond, E. C. Walter, R. Seshadri, L. E. Brus, M. L. Steigerwald, *Journal of the American Chemical Society* **2008**, *130* (22), 6955.

[5] L. You, F. Liu, H. Li, Y. Hu, S. Zhou, L. Chang, Y. Zhou, Q. Fu, G. Yuan, S. Dong, *Advanced Materials* **2018**, *30* (51), 1803249.

[6] H. Y. Ye, W. Q. Liao, C. L. Hu, Y. Zhang, Y. M. You, J. G. Mao, P. F. Li, R. G. Xiong, *Advanced Materials* **2016**, *28* (13), 2579.

[7] Z. Sun, X. Liu, T. Khan, C. Ji, M. A. Asghar, S. Zhao, L. Li, M. Hong, J. Luo, *Angewandte Chemie International Edition* **2016**, *55* (22), 6545.

[8] L. You, F. Liu, H. Li, Y. Hu, S. Zhou, L. Chang, Y. Zhou, Q. Fu, G. Yuan, S. Dong, H. J. Fan, A. Gruverman, Z. Liu, J. Wang, *Advanced Materials* **2018**, *30* (51).

[9] W.-Q. Liao, Y. Zhang, C.-L. Hu, J.-G. Mao, H.-Y. Ye, P.-F. Li, S. D. Huang, R.-G. Xiong, *Nature Communications* **2015**, *6* (1), 7338.

[10] X. Liu, Z. Wu, T. Guan, H. Jiang, P. Long, X. Li, C. Ji, S. Chen, Z. Sun, J. Luo, *Nature Communications* **2021**, *12* (1), 5502.

[11] C.-R. Huang, X. Luo, X.-G. Chen, X.-J. Song, Z.-X. Zhang, R.-G. Xiong, *National Science Review* **2020**, *8* (5).

[12] T. T. Sha, Y. A. Xiong, Q. Pan, X. G. Chen, X. J. Song, J. Yao, S. R. Miao, Z. Y. Jing, Z. J. Feng, Y. M. You, R. G. Xiong, *Advanced Materials* **2019**, *31* (30), e1901843.

[13] C. Ji, D. Dey, Y. Peng, X. Liu, L. Li, J. Luo, *Angewandte Chemie* **2020**, *132* (43), 19095.

[14] C. Ji, Y. Li, X. Liu, Y. Wang, T. Zhu, Q. Chen, L. Li, S. Wang, J. Luo, *Angewandte Chemie International Edition* **2021**, *60* (38), 20970.

[15] L. Li, X. Shang, S. Wang, N. Dong, C. Ji, X. Chen, S. Zhao, J. Wang, Z. Sun, M. Hong, *Journal of the American Chemical Society* **2018**, *140* (22), 6806.

[16] Z. Li, Y. Wang, G. Tian, P. Li, L. Zhao, F. Zhang, J. Yao, H. Fan, X. Song, D. Chen, Z. Fan, M. Qin, M. Zeng, Z. Zhang, X. Lu, S. Hu, C. Lei, Q. Zhu, J. Li, X. Gao, J.-M. Liu, *Science Advances* **2017**, *3* (8), e1700919.

[17] L. Han, C. Addiego, S. Prokhorenko, M. Wang, H. Fu, Y. Nahas, X. Yan, S. Cai, T. Wei, Y. Fang, H. Liu, D. Ji, W. Guo, Z. Gu, Y. Yang, P. Wang, L. Bellaiche, Y. Chen, D. Wu, Y. Nie, X. Pan, *Nature* **2022**, *603* (7899), 63.

[18] J. Ma, J. Ma, Q. Zhang, R. Peng, J. Wang, C. Liu, M. Wang, N. Li, M. Chen, X. Cheng, P. Gao, L. Gu, L.-Q. Chen, P. Yu, J. Zhang, C.-W. Nan, *Nature Nanotechnology* **2018**, *13* (10), 947.
